# Investigating the effect of template head models on Event-Related Potential source localization: a simulation and real-data study

**DOI:** 10.3389/fnins.2024.1443752

**Published:** 2024-10-08

**Authors:** Emma Depuydt, Yana Criel, Miet De Letter, Pieter van Mierlo

**Affiliations:** ^1^Medical Imaging and Signal Processing Group, Department of Electronics and Information Systems, Ghent University, Ghent, Belgium; ^2^BrainComm Research Group, Department of Rehabilitation Sciences, Ghent University, Ghent, Belgium

**Keywords:** Event-Related Potentials, ERP, EEG, source estimation, EEG source localization, head modeling

## Abstract

**Introduction:**

Event-Related Potentials (ERPs) are valuable for studying brain activity with millisecond-level temporal resolution. While the temporal resolution of this technique is excellent, the spatial resolution is limited. Source localization aims to identify the brain regions generating the EEG data, thus increasing the spatial resolution, but its accuracy depends heavily on the head model used. This study compares the performance of subject-specific and template-based head models in both simulated and real-world ERP localization tasks.

**Methods:**

Simulated data mimicking realistic ERPs was created to evaluate the impact of head model choice systematically, after which subject-specific and template-based head models were used for the reconstruction of the data. The different modeling approaches were also applied to a face recognition dataset.

**Results:**

The results indicate that the template models capture the simulated activity less accurately, producing more spurious sources and identifying less true sources correctly. Furthermore, the results show that while creating more accurate and detailed head models is beneficial for the localization accuracy when using subject-specific head models, this is less the case for template head models. The main N170 source of the face recognition dataset was correctly localized to the fusiform gyrus, a known face processing area, using the subject-specific models. Apart from the fusiform gyrus, the template models also reconstructed several other sources, illustrating the localization inaccuracies.

**Discussion:**

While template models allow researchers to investigate the neural generators of ERP components when no subject-specific MRIs are available, it could lead to misinterpretations. Therefore, it is important to consider a priori knowledge and hypotheses when interpreting results obtained with template head models, acknowledging potential localization errors.

## 1 Introduction

Electroencephalography (EEG) is an essential tool for analyzing brain activity, which allows researchers to study the neuronal mechanisms at work when executing specific tasks at a millisecond scale (Luck, 2014). While this technique offers excellent temporal resolution, its spatial resolution is limited, as the signals are measured at the scalp using a limited number of electrodes. Moreover, due to volume conduction, the activity recorded by each electrode does not represent a single underlying brain source, but rather a composite of activities from various brain regions, again limiting the spatial accuracy of EEG. EEG source imaging was introduced to overcome this limitation as a computational technique to estimate the electrical neuronal activity in the brain. This technique identifies the underlying generators of the electrophysiological activity recorded at the scalp by combining the EEG signals with structural MR images. During recent decades, EEG Source Imaging (ESI) has been an important area of research. However, while it has introduced significant advances in multiple research domains such as epilepsy (Mégevand and Seeck, 2020) and sleep (Del Felice et al., 2014; Fernandez Guerrero and Achermann, 2019), the precise localization of the neuronal activity is still a challenge, and the spatial resolution remains unclear.

Source analysis of EEG data consists of two different processes, namely a forward model and an inverse model. The forward or head model describes how a known source of electrical activity within the brain contributes to the signal observed at each EEG electrode on the scalp. The inverse model then estimates the location and the strength of the electrical activity within the brain based on the EEG signals recorded at the scalp, and relies on the forward model to obtain an accurate solution. As this is a non-unique problem, regularization techniques or constraints are needed to find plausible solutions. Many different techniques have been proposed for solving the inverse problem, such as single dipole models, multiple dipole models, including multiple emitter location and signal parameter estimation (MUSIC) (Schmidt, 1986), and distributed source estimation methods, including the minimum norm estimate (MNE), dynamic statistical parametric mapping (dSPM), standardized low-resolution brain electromagnetic tomography (sLORETA) and exact low-resolution brain electromagnetic tomography (eLORETA) (Hämäläinen and Ilmoniemi, 1994; Dale et al., 2000; Pascual-Marqui, 2002; Pascual-Marqui et al., 2011). However, the accuracy of the EEG reconstruction obtained with each of these techniques still depends on the accuracy of the forward model.

The construction of the forward model is thus a critical step in the source reconstruction. The model takes into account the anatomical structure of the head, as well as the electrical conductivity of the different tissue types. Many different studies have investigated the effect of the head model on the obtained localization errors. Vorwerk et al. (2012) and Birot et al. (2014), for example, have investigated the effect of using different methods for the creation of the head model, such as boundary element models (BEMs), finite difference models (FDMs) or finite element models (FEMs). In other studies, the influence of including more head tissue compartments in the model was investigated (Vorwerk et al., 2014; Neugebauer et al., 2017). Recently, Nielsen et al. (2023) specifically investigated the influence of anatomical accuracy and electrode positions on the accuracy of the forward solutions. Other work by Montes-Restrepo et al. (2014) and Montes-Restrepo et al. (2016), for example, studied the influence of different skull modeling approaches on EEG source localization, while Stenroos and Hauk (2013) looked into the robustness of source estimation in the case of skull conductivity errors. Also the influence of head tissue conductivity uncertainties on dipole reconstructions has been investigated (Vorwerk et al., 2019), as McCann et al. (2019) have shown that the electrical conductivity values assumed for each compartment likely vary between individuals. It is clear that the ideal head model for the most accurate reconstruction of the neural activity is a realistic head model created using the subject's individual MRI and accurate electrical properties of the different tissue types (Akalin Acar and Makeig, 2013; Vorwerk et al., 2018; Conte and Richards, 2021).

Unfortunately, in many EEG studies the additional acquisition of MRI data proves difficult. The acquisition of MRI data for each subject would require more time, research funds, and the availability of an MRI scanner. Therefore, many studies using ESI to source localize ERP data use an approximate, average or template-based head model (Sabeti et al., 2016; Dorme et al., 2023; Criel et al., 2024). The effect of this simplification has been studied extensively before. Valdés-Hernández et al. (2009), for example, investigated the performance of approximate models of the head in ESI using simulations and showed that the average of many individual MRI-based models outperforms a randomly selected individual model. Liu et al. (2023) quantified source localization discrepancies introduced by using template head models, inexact electrode locations, and inaccurate skull conductivity for both younger and older adults using real EEG data. They found that using template MRIs led to localization discrepancies of up to 2 cm compared to the anatomically accurate subject-specific head models for both younger and older adults.

However, most studies investigating EEG source localization accuracy have focused on the localization of a single source and quantified the localization error associated with each source modeled within the brain (Vorwerk et al., 2014; Hauk et al., 2022). This approach is motivated by applications in which the activity is dominated by a single source, e.g. in the localization of epileptiform interictal discharges. However, it is known that in multiple applications of ESI, such as Event-Related Potential (ERP) research, typically more than one source is involved in the observed waveform, as more than one brain region is involved in processing the stimuli. It is therefore important to investigate the effect of the head model that is used particularly when multiple sources of activity are present. In a study by Cho et al. (2015), the influence of imperfect head models on EEG source connectivity analyses has been studied with multi-source scenarios, where they found that neglecting the distinction between gray and white matter or neglecting CSF causes large connectivity errors. However, they only used a single subject in this study, and they did not yet investigate the effect of using a template head model.

The objective of this study is therefore to investigate the effect of using a template head model instead of subject-specific head models, particularly in the context of Event-Related Potentials (ERPs) involving multiple brain regions, and to quantify the localization error associated with this simplification. By using both simulated and real task data, the aim is to quantify the localization errors introduced by this simplification and assess the interpretability of the reconstructed neural activity.

## 2 Materials and methods

### 2.1 Participants and data

In this work, the open-source multimodal neuroimaging dataset VEPCON (OpenNeuro Dataset ds003505) was used (Pascucci et al., 2022), in which visual evoked potentials were recorded while the subjects discriminated faces from scrambled faces. This dataset has previously been used in different studies, for example, to improve and validate EEG source imaging methods and time-varying functional connectivity methods (Rubega et al., 2019; Pascucci et al., 2020). The dataset includes raw data, derivatives of high-density EEG, structural MRI and diffusion-weighted images (DWI), and single-trial behavior.

The dataset includes the data of twenty participants (3 males, mean age = 23 ± 3.5) who were recruited from the student population at the University of Fribourg, Switzerland. In this work, only the raw high-density EEG, recorded during a face detection task, and the derivatives of the T1-weighted structural MRI data, obtained using the Freesurfer software, were used. Subjects for whom (part of) this data was missing were excluded, resulting in a total of eighteen participants. The EEG data were recorded at a sampling rate of 2048 Hz with a 128-channel Biosemi Active Two EEG system (Biosemi, Amsterdam, The Netherlands) in a dimly lit and electrically shielded room. More information regarding the dataset and recording procedures can be found in the data descriptor provided by Pascucci et al. (2022).

### 2.2 MRI processing and head model reconstruction

Preprocessed structural MRI data was included in the open-source dataset. For each subject, Pascucci et al. (2022) resampled the T1w images using the Connectome Mapper v3.0.0-beta-RC1 pipeline (Tourbier et al., 2022), and segmented gray and white matter using Freesurfer 6.0.1 (Fischl, 2012). The structures were then parcellated into 83 cortical and subcortical areas according to the Desikan-atlas. Also other parcellations were included in the dataset, such as the parcellation following the Destrieux atlas.

Multiple approaches were used for the construction of the forward model, namely the finite element method (FEM) and the boundary element method (BEM). The FEM method uses a realistic volume mesh of the head, which is generated from the MRI segmentation, and results in anatomically accurate models. The BEM model, on the other hand, relies on the creation of three BEM surfaces (inner skull, outer skull, and skin) and thus includes less detailed segmentations in the model.

#### 2.2.1 FEM

A finite element method (FEM) head model was constructed for each subject in Brainstorm (Tadel et al., 2011), which is documented and freely available for download online under the GNU general public license. In the first step, the tetrahedral FEM meshes were generated using the SimNIBS-charm pipeline (Puonti et al., 2020). The MRI data was segmented into nine different tissue types: white matter, gray matter, CSF, compact bone, spongy bone, scalp, eyes, blood and muscle, after which the meshes representing the geometry of the head were created. Equivalent current dipoles were then distributed within the gray matter. The dipoles were spaced approximately 3 mm apart, resulting in a dense and uniform grid of dipoles throughout the cortical surface. The forward model was subsequently generated from the obtained mesh using the DUNEuro-FEM computation within Brainstorm (Medani et al., 2023).

Two different forward models were created based on the FEM meshes. In the first model, the conductivity values for the different tissue types were based on the weighted average means from the meta-analysis by McCann et al. (2019): 0.22 S/m for white matter, 0.47 S/m for gray matter, 1.71 S/m for the CSF, 0.006 S/m for the compact bone and 0.048 S/m for the spongiform bone, 0.41 S/m for the scalp, 0.33 S/m for the eyes, 0.57 S/m for blood and finally 0.32 S/m for the muscle layer. In the second model, the default conductivity values as proposed by Brainstorm were used: 0.14 S/m for white matter, 0.33 S/m for gray matter, 1.79 S/m for the CSF, 0.008 S/m for the compact bone and 0.025 S/m for the spongiform bone, 0.43 S/m for the scalp, 0.33 S/m for the eyes, 0.33 S/m for blood and 0.33 S/m for the muscle layer (Vorwerk et al., 2014). By including two models with different conductivity values, it is possible to investigate the effect of using slightly deviant conductivities on the reconstructions.

In addition to individual head models, the same approach was applied to the average MRI, fsaverage, available in Freesurfer. This template brain is based on a combination of 40 MRI scans of real brains. More information on the creation of the fsaverage template and details about the subjects used in this template can be found in the official Freesurfer documentation (Fischl, 2012).

#### 2.2.2 BEM

For each individual, a three-layered head model was created using Freesurfer 6.0.1 and MNE-python (Fischl, 2012; Gramfort, 2013). The inner skull, outer skull and outer skin surfaces were obtained from the dataset and then used as boundaries for the different compartments, assigning default electrical conductivity values to the scalp (0.33 S/m), skull (0.006 S/m) and brain (0.33 S/m) compartments of the head model. The same equivalent current dipole locations as used in the FEM models were used here, i.e. the dipoles were distributed in the gray matter with a spacing of 3 mm. Finally, the boundary element method (BEM) was used to obtain the EEG leadfield matrix. As before, this approach was also applied to the average MRI, fsaverage, to obtain the leadfield matrix for the average head model.

### 2.3 ERP preprocessing

The high-density EEG data recorded during the face recognition task was processed using the MNE-python library (Gramfort, 2013). The data were first downsampled to 250 Hz and bad electrode channels were automatically detected using the different noisy channel detection methods in the PREP pipeline (Bigdely-Shamlo et al., 2015). The electrodes indicated as bad were excluded from further analysis. The data was band-pass filtered using a zero phase shift Butterworth filter with half-amplitude cut-off frequencies of 0.3 Hz and 30 Hz and a 12 dB/octave slope. The power line noise was then removed using a 50 Hz notch filter. Independent component analysis was applied for eye blink and horizontal eye movement artifact rejection. In case bad electrode channels were identified and excluded in the first step, these channels were interpolated at this stage. Subsequently, data were re-referenced to an average common reference. In the next step, the data was segmented into epochs going from 100 ms before the stimulus onset to 500 ms after. Finally, epochs containing artifacts were rejected using the following criteria: 75 μV maximum gradient criterion; 100 μV minimal/maximal amplitude criterion; 150 μV maximum difference criterion; 0.5 μV low activity criterion during 100 ms.

### 2.4 Simulation

Simple ERP waveforms were simulated using half-cycle sinusoidal waveforms to allow the objective quantification of the localization error associated with the subject-specific and average head models. This was done by simulating activity in different regions of the brain, including noise, and projecting this activity to the scalp surface using the individual head models. For each subject, 80 epochs of 1000 ms were simulated, half of which contained the ERP waveform as well as pink noise, while only the noise was included in the other half. In each epoch, a pre-stimulus window of 200 ms was considered. By including epochs which only contain noise, and thus simulating two different conditions, it is possible to investigate the difference between the localizations obtained for both conditions. This approach helps in reducing systematic biases in the source reconstruction process. If certain types of noise or non-specific activity consistently affect the EEG data, this might lead to similar localization errors across both conditions. By subtracting one condition from another, these systematic errors can be reduced, leading to a more accurate estimate of the neural sources.

Different networks responsible for generating ERP activity were simulated, each involving four symmetrically active brain regions, with two regions in each hemisphere. These regions were identified using the Destrieux cortical atlas parcellations. For each region of interest (ROI), the center of the parcellation was determined, and dipoles within a 10 mm radius around this center were selected. The ERP activity in these selected dipoles was simulated as a 5 Hz half-cycle sinusoidal waveform lasting 100 ms. A small delay was introduced across the ROIs: the ERP waveform began in the first ROI at 100 ms post-stimulus, followed by the second ROI 10 ms later, and then in the third and fourth ROIs at 120 ms. Additionally, the signal amplitude in the third and fourth ROIs was reduced to 80% of the amplitude in the first two ROIs. Table 1 provides an overview of the different ROIs selected for each network. These networks were designed to investigate localization errors across different ROIs, as previous studies have shown that localization errors are typically larger for temporal sources (Cuffin et al., 2001; Kobayashi et al., 2003). To simulate realistic conditions, pink noise was added to all epochs. The noise amplitude was adjusted to achieve different signal-to-noise ratios (SNRs) ranging from -20 dB to +0 dB. The SNR was defined as the ratio of the peak amplitude of the ERP component to the peak-to-peak amplitude measured within the pre-stimulus window. This SNR-range was chosen based on the VEPCON dataset, where an SNR of about -10 dB was observed for the N170 component.

**Table 1:** Overview of the different ROIs used for the simulation of the networks.

**Network**	**ROI 1**	**ROI 2**	**ROI 3**	**ROI 4**
Temporo-occipital network	Occipital pole (lh)	Occipital pole (rh)	Inferior temporal sulcus (lh)	Inferior temporal sulcus (rh)
Fronto-parietal network	Inferior frontal gyrus, pars opercularis (rh)	Inferior frontal gyrus, pars opercularis (lh)	Supramarginal gyrus (lh)	Supramarginal gyrus (rh)
Fronto-occipital network	Occipital pole (lh)	Occipital pole (rh)	Inferior frontal gyrus, pars opercularis (rh)	Inferior frontal gyrus, pars opercularis (lh)
Temporo-parietal network	Inferior temporal sulcus (lh)	Inferior temporal sulcus (rh)	Supramarginal gyrus (lh)	Supramarginal gyrus (rh)

lh, left hemisphere; rh, right hemisphere.

After creating the simulated activity in source space, the source time series were projected to the scalp by applying the subject-specific FEM forward model created using the individual MRI for each subject and using the conductivity values based on the meta-analysis by McCann et al. (2019). This step results in individual epochs in sensor space, or thus the simulated EEG data.

### 2.5 Brain activity reconstruction

For the reconstruction of the brain activity, the MNE-python implementation of the exact Low-Resolution Tomography (eLORETA) inverse method was used (Pascual-Marqui et al., 2011). The source reconstruction was done for each epoch separately, using both the subject-specific head models and the average head models that were previously constructed using the three different modeling pipelines. Noise pre-whitening of the leadfield matrix was applied using the noise covariance matrix before calculating the inverse solution. Next, the absolute magnitude of the dipoles or the current source density (CSD) was calculated, disregarding the orientation information of the dipoles in subsequent analyses. In a final step, for each subject, each condition and for each head model, the average response was calculated. In the case of the simulated data, an evoked response was obtained for the ERP and the noise condition for each subject and both the subject-specific and the average head models, while for the experimental data, an evoked response in source space to the faces and to the scrambled images was obtained, again for each subject and for both the subject-specific and the average head models.

### 2.6 Evaluation of the source reconstruction

#### 2.6.1 Simulated data

Different aspects are taken into account in the evaluation of the source reconstruction: the correspondence between the obtained sources and the simulated sources, the localization error and the spatial dispersion of these reconstructed sources, and the correlation between the originally simulated activity and the reconstructed activity.

For each subject, the difference in source space activity between the ERP and the noise condition is calculated, after which the data is averaged over the time window of interest, in this case from 100 ms to 220 ms post-stimulus. The data is then thresholded so that only the 5% strongest differences between the ERP and noise conditions remain, after which the remaining active dipoles are grouped into potential clusters based on the spatial adjacency. Two dipoles are considered to be adjacent if the distance between both dipoles is smaller than 5 mm. Finally, only clusters containing at least five dipoles are retained.

**Figure 1:**
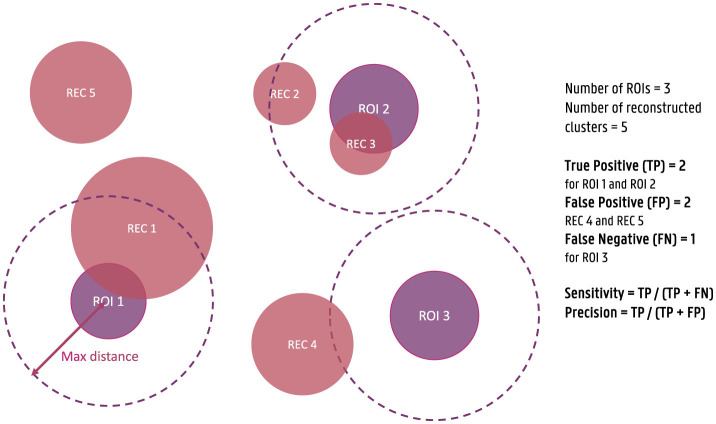
Illustration of the calculation of sensitivity and precision of the source reconstructions for the simulated data. True positives (TPs) are defined as reconstructed clusters within a 3 cm distance from the center of the simulated ROIs, while false negatives (FNs) are ROIs without a nearby cluster, and false positives (FPs) are clusters not within 3 cm of any ROI. Sensitivity is calculated as the ratio of TPs to the sum of TPs and FNs, and precision is calculated as the ratio of TPs to the sum of TPs and FPs.

For each of the obtained dipole clusters, the distance between the center of the cluster and the center of the simulated ROIs is calculated. Each ROI for which at least one reconstructed cluster is found within a 3 cm distance is considered a true positive (TP), while ROIs without a cluster within this distance are considered false negatives (FN). Similarly, clusters that are not within a 3 cm distance of a simulated ROI are annotated as false positives (FP). Based on this classification of the clusters, the sensitivity and the precision of the localization are then calculated as respectively the ratio of the number of TPs over the sum of the TPs and the FNs and the ratio of the number of TPs over the sum of the TPs and FPs. These measures are used to quantify the correctness of the reconstructed activity. To clarify these metrics further, a figure illustrating the calculation of sensitivity and precision is provided (Figure 1). As the maximal distance is an important parameter, also the effect of this parameter was investigated by including the results of using a maximal distance of 1 cm and 5 cm in Appendix.

For the calculation of the localization error and the spatial dispersion, only the true positive ROIs are taken into account. The localization error is calculated as the Euclidean distance between the center of the simulated ROI and the reconstructed cluster. When more than one cluster is within the 3 cm distance of the ROI, the average of the localization errors is taken into account. The spatial dispersion, on the other hand, is calculated as the difference between the total volume of all reconstructed clusters within the 3 cm distance of the ROI and the total volume of the ROI. This measure is then normalized by dividing by the total volume of the simulated ROI to take into account differences in the dispersion of the original activity.

#### 2.6.2 VEPCON data

As no ground truth data exists for the sources underlying the signals measured during the face task, the evaluation of the reconstructed activity can only be evaluated descriptively. One of the ERP components elicited by the faces task that is used in the VEPCON dataset is the N170. This component is larger when the eliciting stimulus is a face compared to when the stimulus is a non-face object, such as a scrambled face or a car [for a review, see Rossion and Jacques (2012)]. Many researchers have investigated the sources underlying this component. Using a dipolar fit method, Taylor et al. (2001) have located the N170 in the middle part of the fusiform gyrus. This localization corresponds to the fusiform face area that was identified in fMRI studies (Haxby et al., 2000), as well as in intracranial EEG studies (Engell and McCarthy, 2014). Similarly, Henson et al. (2007) found differences between the localization of faces and scrambled faces in the anterior fusiform gyrus, with a strong dominance toward the right hemisphere.

In this work, each of the individual epochs will be source-localized using both the subject-specific head models and the average head models. The obtained localization will then be averaged for each condition separately and, in the case the subject-specific head model was used, the obtained results will be morphed to the average head model after which averaging can be applied over all subjects. The 5% dipoles with the strongest difference in activation between the two conditions within a time window of 150 ms to 170 ms post-stimulus will then be visualized and compared to the regions identified in the literature.

## 3 Results

### 3.1 Simulated data

**Figure 2:**
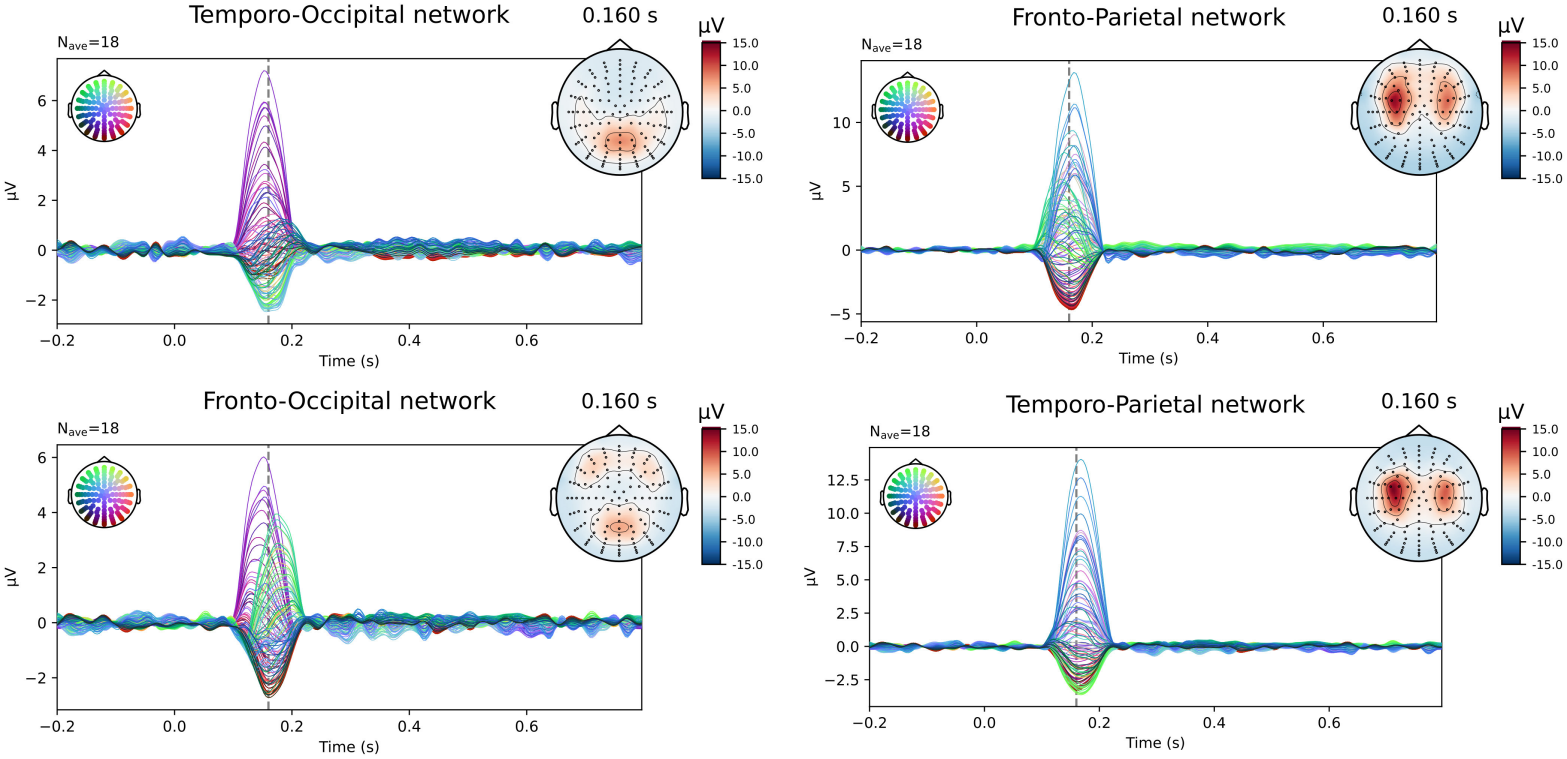
Overview of the simulated data at sensor level averaged over all subjects. The simulated epochs in the ERP condition at SNR = -10 dB are averaged.

The simulated EEG data of the different networks was source localized for each subject using both the subject-specific and the template head models, using both of the FEM models and a BEM model for the reconstruction. Figure 2 shows the simulated data at the sensor level. In the figure, the symmetric nature of the simulated data is visible. While in most networks the activity of the different ROIs of a single hemisphere is blended at the surface, for the fronto-occipital network a clear distinction is visible between the frontal and the occipital subcomponents of the simulated ERP waveform, both when looking at the topography of the obtained signal and when inspecting the waveform. This effect might facilitate the source localization compared to the other networks, where the activity from the different sources is less separated spatially at the scalp level.

**Figure 3:**
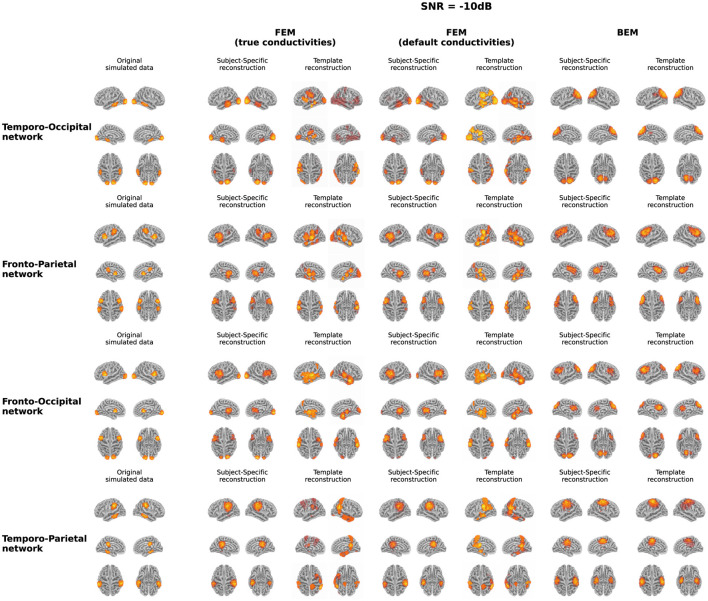
Overview of the original simulated data and the reconstructed activity averaged over all subjects for the different networks at SNR = -10 dB. For the reconstructed activity, the difference between the ERP- and the noise-conditions is shown. In the case of the simulations and the subject-specific reconstructions, the source activity was morphed to the anatomy of the average head model before averaging.

In Figure 3, both the originally simulated data in source space and the reconstructed activity averaged over all subjects for the different networks for an SNR level of -10dB are shown. To reduce systematic biases in the source reconstruction, the difference between the reconstructed activity for the ERP and noise conditions is shown. As averaging over subject-specific anatomies is not possible, the source activity of both the original simulated data and the subject-specific reconstruction was morphed into the anatomy of the average head model before averaging. The figure illustrates the differences between the obtained reconstruction when using the different models. The results obtained with the two FEM models, constructed using different conductivity values, are very similar for most of the networks. While differences in the intensity of the activity can be observed, the location of the activity averaged over all subjects is very similar when using the FEM models with different conductivities. Looking at the different networks, the figures show that the location of the ROI influences on the accuracy of the localization. For the temporo-parietal network, for example, the activity in the temporal lobes is not reconstructed using the subject-specific FEM-based models, while it is clearly present for the temporo-occipital network. The differences between the subject-specific reconstructions and the template reconstructions illustrate that for both FEM approaches better results are obtained using the subject-specific reconstructions. It is clear that while most simulated are reconstructed, i.e., taking into account some mislocalizations, also many false positive clusters are reconstructed. Finally, the figure shows that the results obtained using the BEM models perform quite poorly. Limited differences are found between the subject-specific reconstructions and the template reconstructions in this case in terms of the location of the reconstructed activity. Upward mislocalizations seem to be present for all of the different networks when using BEM models. The occipital sources are localized more toward the superior parietal lobe, for example, while no clear reconstruction can be found for the temporal ROIs. Finally, the figure also shows that only for the fronto-occipital network two distinct ROIs are localized per hemisphere, while only a single spread-out ROIs is reconstructed per hemisphere for the other networks.

**Figure 4:**
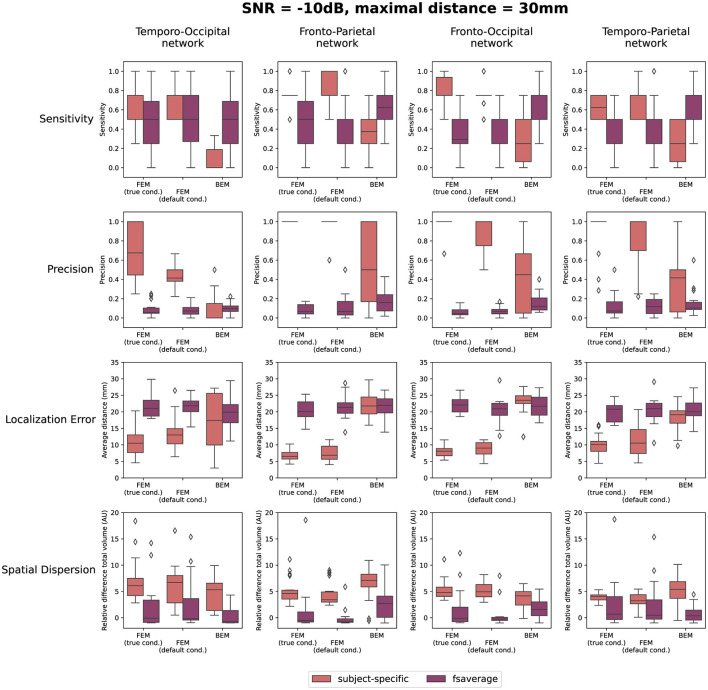
Results of the quantification of the localization errors for the SNR = -10 dB. In this evaluation the sensitivity and the precision of the obtained sources, the localization error and the spatial dispersion of these reconstructed sources were taken into account. For each of these measures, the difference between using the subject-specific and average head models is shown for each of the different modeling approaches. Clusters of activity were considered to be correctly localized when the difference between the center of the reconstructed cluster was within 3 cm of the center of the simulated ROIs.

The quantification results of the localization errors associated with the localizations for the individual subjects are shown in Figure 4. In this evaluation of the source reconstructions, different aspects were taken into account: the sensitivity and the precision of the obtained sources, the localization error and the spatial dispersion of these reconstructed sources. For each of these measures, the difference between using the subject-specific and the average head models was investigated, as well as the differences between the different modeling approaches. Clusters of activity were considered correctly localized when the difference between the center of the reconstructed cluster was within 3 cm of the center of the simulated ROIs. As this maximal distance is an important parameter, the results when using a maximal distance of both 1 cm and 5 cm were included in Appendix. Looking at the different FEM models, higher sensitivity and precision, as well as smaller localization errors were found when using the subject-specific head models compared to the template head model. These trends were found for the different simulated networks, however, some individual differences were observed. For most subject-specific reconstructions, a sensitivity value of about 0.75 is achieved, meaning that one out of the four simulated ROIs was not reconstructed for most subjects. The mean sensitivity obtained using the template-based head models is lower, around 0.5, illustrating that only two ROIs are correctly reconstructed. The sensitivity of the template-based reconstructions however increases for all models when increasing the maximal distance to consider reconstructed activity to 50 mm, indicating that the ROIs are reconstructed with a large localization error. The precision of the localizations is quite high for all of the subject-specific reconstructions, for all networks, indicating that only a limited number of false positive reconstructed sources were found. Very low precision values are found however when using the template head models. This result again illustrates that while the different ROIs are reconstructed, the localization error associated with them is too large to consider them as true positives.

Only considering the clusters located closely to the simulated ROIs, the localization error and the spatial dispersion were investigated. These results are shown in respectively the third and the fourth row of Figure 4. It is clear that for both FEM-based head modeling approaches, the localization error is smaller for subject-specific reconstructions compared to the template reconstructions. Surprisingly, higher spatial dispersion is found when using subject-specific headmodels compared to using the template head models, meaning that larger volumes of reconstructed activity are found compared to the simulated data.

Looking at the BEM models, higher sensitivity values are found when using the template head models compared to the subject-specific head models, while the opposite effect is found for the precision. A large range of precision values is found when using the subject-specific head models, indicating that in this case, the number of false positive reconstructed clusters is very dependent on the individual subject. As expected based on the results shown in Figure 3, similar localization errors are found for the subject-specific and the template-based BEM models. Interesting to note is that the quantitative results for all template-based head models in terms of sensitivity, precision, localization error and spatial dispersion are similar for the two FEM models and the BEM model, while different errors are made in terms of the location of the reconstructed sources (cf. Figure 3).

**Figure 5:**
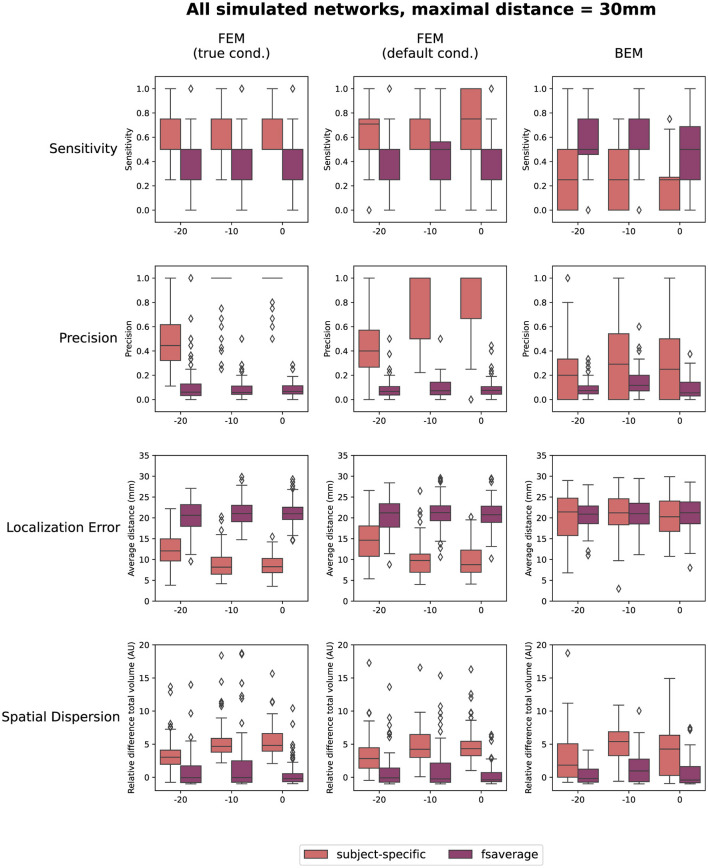
Results of the quantification of the localization errors. In this evaluation the sensitivity and the precision of the obtained sources, the localization error and the spatial dispersion of these reconstructed sources were taken into account. For each of these measures, the effect of both the SNR of the simulated data and the difference between using the subject-specific and average head models is shown for each of the different modeling approaches. Clusters of activity were considered to be correctly localized when the difference between the center of the reconstructed cluster was within 3 cm of the center of the simulated ROIs.

Finally, also the effect of the SNR of the simulated data on the reconstructions is quantified in Figure 5. In this figure, the results obtained for the different networks are aggregated. As before, the results when using a maximal distance of both 1 cm and 5 cm are included in the Supplementary material. The figures indicate only a limited effect of the SNR for most measures. A slight increase in sensitivity with increasing SNR can be found for the subject-specific FEM models, as the boxplots indicate that there are fewer subjects for whom only one or two of the simulated ROIs are reconstructed. Also, an increasing trend with increasing SNR was found when looking at the precision. Finally, a limited improvement can be found in the localization error when increasing the SNR from -20 dB to -10 dB. Further increase of the SNR has almost no effect. The most prominent conclusions that can be drawn from this figure, however, are again that for both FEM-based modeling approaches, the subject-specific head models perform better than the template-based methods in terms of sensitivity, precision and localization errors, while the BEM-based modeling approaches perform worse in the case of subject-specific models but perform similarly when using template-based models compared to the FEM-models.

### 3.2 Real task data

**Figure 6:**
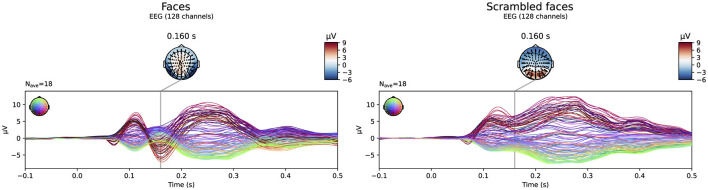
Visualization of the evoked potentials averaged over all subjects for both the faces and scrambled faces conditions of the face-detection task in the VEPCON dataset. Also the topography at 160 ms post-stimulus is indicated, as this is considered the peak of the N170 component in the faces condition.

**Figure 7:**
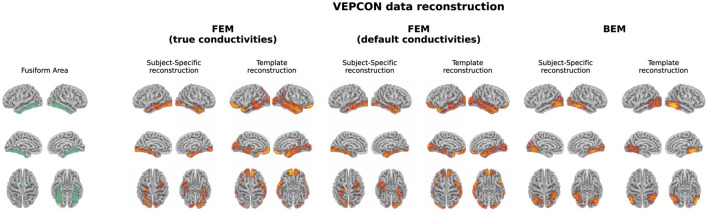
Illustration of the fusiform area and the difference in the reconstructed activity between both conditions for the N170 component averaged over all subjects. For the subject-specific reconstructions, the source activity was morphed to the template head model before averaging. In the case of the subject-specific head models using FEM-based approaches, activity is found in the left and right fusiform area. When using the template head models, on the other hand, the strongest difference in activity between both conditions is found more occipitally.

The evoked potentials averaged over all subjects are shown in Figure 6 both for the faces and the scrambled faces. A clear difference between both conditions was found between 150 ms and 170 ms after the stimulus onset. The N170 component is thus clearly present in the data when faces were presented to the subjects, while it is not in the scrambled faces condition. Figure 7 shows the difference of the obtained reconstructions between both conditions averaged over all subjects using both the subject-specific head models and the template head model for the different head modeling approaches. In the first column of the figure also the expected reconstructed area, i.e. the fusiform area, is shown. The figures show that in the case of the subject-specific FEM head models, most activity is found in the left and right fusiform area while using the subject-specific BEM model, most activity is found more occipitally. When using the template head model, on the other hand, the reconstructed activity is more spread out compared to the subject-specific reconstructions. In the case of the FEM-models, activity is found not only in the fusiform area, but also at the frontal and temporal poles as well as in occipital lobe. In case of the template BEM models, the largest differences in activity between both conditions are again found occipitally.

## 4 Discussion

The goal of this work was to investigate the effect of using template head models instead of subject-specific head models when localizing ERPs and to quantify the localization error associated with this simplification. To this end, both simulated data and real task data were used. Different activity networks were simulated, each with four ROIs and specific SNRs using subject-specific head models created using FEM. We found that subject-specific head models perform significantly better than template head models, and that the modeling approach (FEM or BEM) only has a limited influence on the accuracy of the results when using template head models.

### 4.1 Simulated data

#### 4.1.1 Effect of template head model vs. subject-specific models

Looking at the simulations, the results indicate there is a significant decrease in both the sensitivity and precision when using template head models instead of subject-specific head models when using FEM-based head models. Interestingly, when using BEM models the sensitivity is better when using the template head model. Also, clear differences in the localization error were found between the subject-specific and the template FEM models, as values between ±5-20 mm are found using the subject-specific headmodels compared to localization errors between ±15-30 mm are found for the template-based models. These results correspond with our hypothesis and with results found in literature, as many researchers report that the ideal head model for the most accurate reconstruction of the neural activity is a realistic head model created using the subject's individual MRI (Akalin Acar and Makeig, 2013; Conte and Richards, 2021). However, it is also important to note that, as subject-specific models were used in the simulations, this is also the case for which the best results were expected.

Looking at the results at the group level (Figure 3), it is clear that not all simulated ROIs are present, especially when using template-based models, and that a localization error is associated with other reconstructed ROIs. As in most ERP research, the MRI data of individual subjects is not available, it is important to take these limitations into account in the interpretation of obtained results. In cases where no subject-specific data is available, it might be helpful to use a hypothesis-driven approach to investigate the cortical generators of a certain ERP component. This approach can help in identifying FPs and possible FNs in the reconstructed sources.

#### 4.1.2 Effect of using different conductivity values

As mentioned in the introduction, studies have shown that accurate electrical conductivity values for the different tissue types included in the head model are important for accurate source localization of the EEG signals (Vorwerk et al., 2019). Furthermore, McCann et al. (2019) have shown that the electrical conductivities assumed for each compartment likely vary between individuals. As the measurement of the electrical conductivities of the different tissue types in individuals is not feasible, the conductivity values used in the created head models will thus always be (slightly) off. To investigate the effect of this error on the localization accuracy, two different FEM models with different conductivity values assigned to the tissues were used in this work. Differences were found in all of the measures used in the quantification of the results with the models using the “true” conductivities giving better results. These found differences are however small, both when the subject-specific and the template-based head models are used.

Furthermore, it should be noted that the subject-specific FEM model using the “true” conductivities was also used in the simulation of the data. This model was thus also expected to yield the best outcome, as an identical transformation was applied to reconstruct the data. Differences between the simulated data and the reconstructed activity in this case can thus be attributed to the assumptions made by the inverse solutions, as this is a non-unique problem. These results thus indicate that, while it is important to use the most accurate conductivity values possible, the effect of deviations in these values is much smaller than the effect of using subject-specific vs. template-based head models.

#### 4.1.3 Effect of FEM vs. BEM models

In this work, different head modeling approaches were used for the reconstruction of the simulated data, namely two FEM models with different conductivity values and a BEM model. As discussed in the previous section, the effect of using different conductivity values for the different tissue types in the FEM models is more limited than the use of subject-specific head models. However, much larger differences are found between the results obtained using the FEM-based head models and the results using the BEM-based models. Looking at the subject-specific reconstruction, the FEM-based models perform better than the BEM-based models across all measures. This result was expected, as BEM models are much less accurate than the FEM-based models that were used because they are unable to take into account cerebrospinal fluid (CSF). Also the influence of including more head tissue compartments in the model has been studied extensively before (Vorwerk et al., 2014; Neugebauer et al., 2017). However, again it is also important to note that, as FEM models were used in the simulations, this is also the case for which the best results were expected.

It is however interesting to note that the quantitative results obtained using the template-based head models are similar across the different modeling approaches. For some networks, the template-based BEM models even perform slightly better than the template-based FEM models in terms of sensitivity. These quantitative results were not what was expected based on the results that were plotted at the group level (Figure 3), where the localization errors seem larger for the template-based BEM models compared to the FEM-models. Combining these results indicates that the localization errors made using the template-BEM model are less systematic than those made using the template-FEM models, i.e., the localizations and the associated errors that are obtained at the individual level are more random than in the case of the FEM models. This will cause some of the reconstructed ROIs to cancel out at the group level, seemingly indicating that these ROIs were not reconstructed using these models.

#### 4.1.4 Effect of the signal-to-noise ratio

While the effect of the SNR is limited in this simulated dataset, some differences are still observed between the lowest simulated SNR, -20 dB, and the other SNR levels. For the subject-specific FEM models, for example, lower precision, higher localization errors and less spatial dispersion are found for the lowest SNR level compared to the others, indicating that more FPs are found for the lower SNRs. It is important to take these results into account when interpreting findings obtained from real data. In the case of low SNRs, multiple sources may be reconstructed which are not related to the true underlying activity of the ERP, even after averaging over all subjects (cf. Figures S3 to S6 in Appendix). Another possibility is that due to the larger localization errors and smaller reconstructed volumes, the obtained sources are not pertained after averaging over all subjects, giving the impression that this source is not present in the data. The differences between the averaged results and the quantifications illustrate the importance of also looking at the results of the reconstruction at the level of the individual subject. Furthermore, these results again illustrate that using a hypothesis-driven approach for interpreting the findings can help. Other methods to increase the SNR of the data might be useful, such as using averaged data instead of individual trials when possible.

However, more and more interest is found in functional connectivity analysis in source space to investigate the networks underlying brain activity. As many functional connectivity measures focus on spectral features of the data, in this case, a priori averaging of the epochs is not possible, as high-frequency information would be averaged out in the data, as well as time- but not phase-locked activity (Simoes et al., 2003). While researchers have already investigated the influence of the head model in terms of neglecting white/gray matter distinction or CSF on EEG source connectivity analyses (Cho et al., 2015), this work shows that also the SNR of the data should be taken into account.

#### 4.1.5 Effect of different networks

Finally, the results show some differences in localization performance for the different networks that were simulated. Looking at the averaged reconstructions, it is clear that not all ROIs were reconstructed for the different networks. When using the template-based FEM models for the reconstruction of the fronto-parietal and the fronto-occipital networks, no frontal ROIS were found in the right hemisphere in the averaged reconstructions. However, the quantitative results found for these networks indicate mean sensitivity values of 0.75. One possible explanation for the absence of the frontal sources could be the reduced amplitude of the simulated signals in these ROIs compared to the signals in the respectively parietal and occipital sources.

Looking at the results obtained using the BEM-based head models, large localization errors were found across the different networks. For all networks except the fronto-occipital network, the different ROIs appear to have been localized as single clusters per hemisphere, indicating that the BEM-based models have more difficulty separating sources of activity. This seems probable, as in the simulated ERP-data at the sensor level, also the fronto-occipital network is the only network in which a clear separation of the underlying sources could be seen in the topography and the waveform of the obtained ERP. Furthermore, for the networks including occipital sources, an upward displacement of these sources could be seen when using the BEM-based head models. Similar mislocalization results were also found by Huang et al. (2016) and Akalin Acar and Makeig (2013), who both identified larger localization errors for occipital sources when using less accurate head models. Multiple explanations can be found in literature for these errors in the localization of occipital sources. The occipital lobes are complex structures with many folds and curves with significant inter-subject variability. Using less accurate head models in this case can thus increase the mislocalization of the sources. In addition to this, also the occipital bone is in general thicker with again significant inter-subject variability. Modeling this using a non-accurate head model will again lead to larger errors in the head model, reducing the localization precision (Michel and Brunet, 2019).

A limitation of this study was that only a limited number of ROIs were investigated. As it was shown that the underlying sources influence the accuracy of the reconstruction, in future work, a more generalized approach should be developed to investigate the effect of different networks more systematically. While such approaches have already been proposed for focal sources (Samuelsson et al., 2021), this problem is much more challenging when considering simultaneous activations and remains, to the best of our knowledge, currently unsolved.

### 4.2 Real task data

Localization of the high-density EEG data in the VEPCON dataset, recorded while presenting faces and scrambled faces to subjects, resulted in different sources using the subject-specific head models and the average head model. Using the subject-specific FEM head models, the N170 component was mainly localized to the left and right fusiform areas. These results correspond to the sources found in other studies (Rossion and Jacques, 2012), both using EEG/MEG data (Henson et al., 2007) and fMRI (Haxby et al., 2000). While the fusiform area is considered the core generator of the N170, there is evidence suggesting that also the prefrontal cortex plays a role in the processing of faces and that this region contributes to the top-down modulation of face processing (Kornblith and Tsao, 2017; Gazzaniga et al., 2009). There was however no activation found in this region in this work.

Looking at the results obtained using the template FEM models, again the left and right fusiform areas were found as generating sources of the N170. However in this case, also multiple other sources were found, such as the frontal and temporal pole and the occipital lobe. Finally, using both the subject-specific and the template-based BEM models, the N170 component was localized more in the occipital lobe with the activity extending toward the posterior inferior temporal lobe, rather than in the fusiform area. As the results obtained using the subject-specific FEM head models correspond well to results reported in the literature, these results indicate that while the template-based head model can be used for the localization of ERP sources, interpretations should be done with care, as mislocalizations of the sources and localization errors can be present. These results show the importance of taking into account hypotheses and a priori knowledge when interpreting the source localization results obtained using a template head model.

## 5 Conclusion

In this study, the effect of using template head models instead of subject-specific head models was investigated in localizing event-related potentials (ERPs) and in quantifying the associated localization error using both simulated and real data. As expected, the results indicated that subject-specific head models outperform template head models in terms of localization accuracy. Using template head models also increases both false positives and false negatives in source reconstructions. Also the effect of using more accurate FEM models compared to simple BEM models was investigated. As found in previous studies, more anatomically accurate head models result in better localization performance. When template-based head models are used however, similar quantitative results in terms of sensitivity, precision, localization error and spatial dispersion were found for the FEM- and BEM-based head models, even though the patterns of mislocalizations are different. Furthermore, the role of the SNR on the localization performance was investigated, with the results showing that low SNRs may lead to larger errors. Finally, the influence of the simulated network also has a significant effect on the accuracy of the source localization, with the results indicating that some regions, such as the temporal and occipital lobes are more prone to mislocalization when using template head models.

While template head models offer a practical alternative for ERP source localization when no subject-specific MRI data is available, their limitations should be considered, and the results should be interpreted with caution. A priori knowledge and hypothesis-driven approaches are crucial for interpreting results obtained with average head models. Interestingly, however, is that while creating more accurate and detailed head models is beneficial for the localization accuracy when using subject-specific head models, this is not the case for template head models. As many studies investigating the effect of modeling approaches for ESI focus on focal sources, it would be beneficial if systematic approaches to assess the influence of multiple sources on localization accuracy would become more prominent.

## Data Availability

Publicly available datasets were analyzed in this study. This data can be found here: doi: 10.18112/openneuro.ds003505.v1.1.1.
