# Supplementary material for: Investigating the effect of template head models on Event-Related Potential source localization: a simulation and real-data study
Source: Front Neurosci. 2024 Oct 8;18:1443752. doi: 10.3389/fnins.2024.1443752 (PMC11493687; doi:10.3389/fnins.2024.1443752)
Supplement: Supplementary file 1 [file Data_Sheet_1.pdf]

Supplementary Material

APPENDIX A: SIMULATED EPOCHS FOR OF THE DIFFERENT NETWORKS FOR ALL SNRS

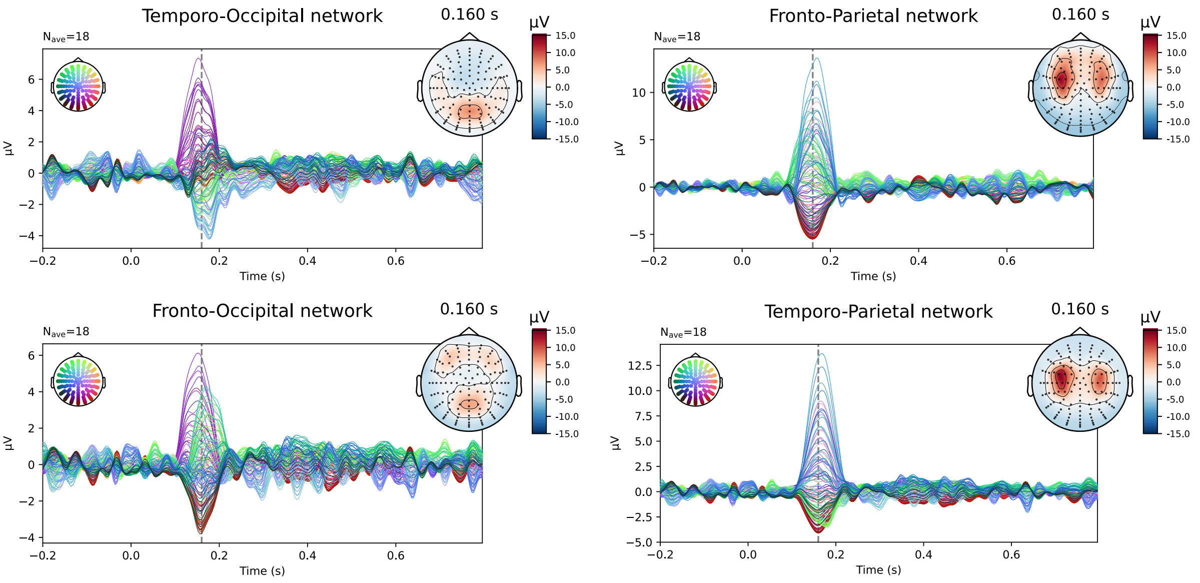

Figure S1. Overview of the simulated data at sensor level averaged over all subjects. The simulated epochs in the ERP condition at SNR = -20dB are averaged.

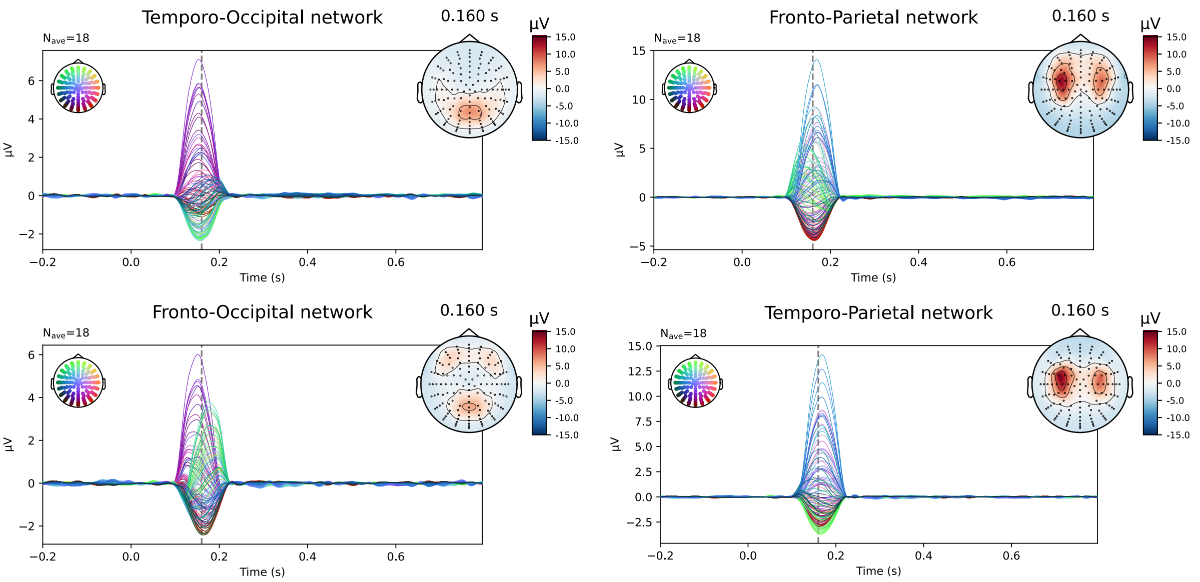

Figure S2. Overview of the simulated data at sensor level averaged over all subjects. The simulated epochs in the ERP condition at SNR = 0dB are averaged.

APPENDIX B: LOCALIZATION OF THE DIFFERENT NETWORKS FOR ALL SIMULATED SNRS

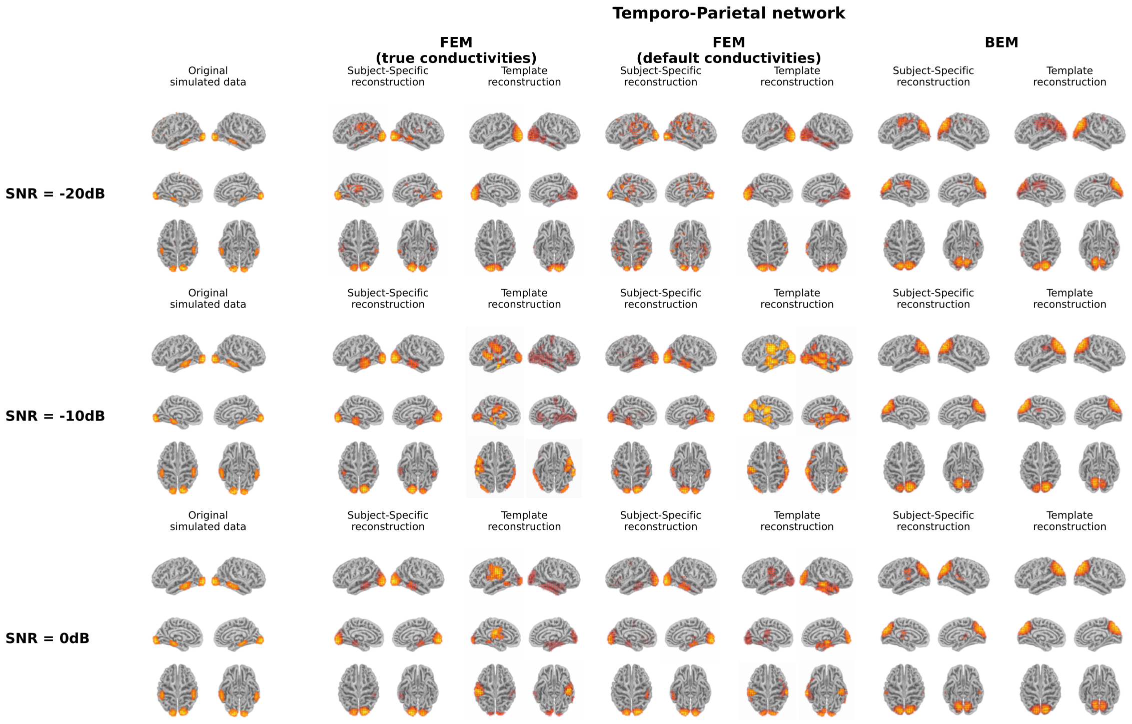

Figure S3. Overview of the original simulated data and the reconstructed activity averaged over all subjects for the temporo-occipital network at the different SNRs. In the case of the simulations and the subject-specific reconstructions, the source activity was morphed to the average head model before averaging.

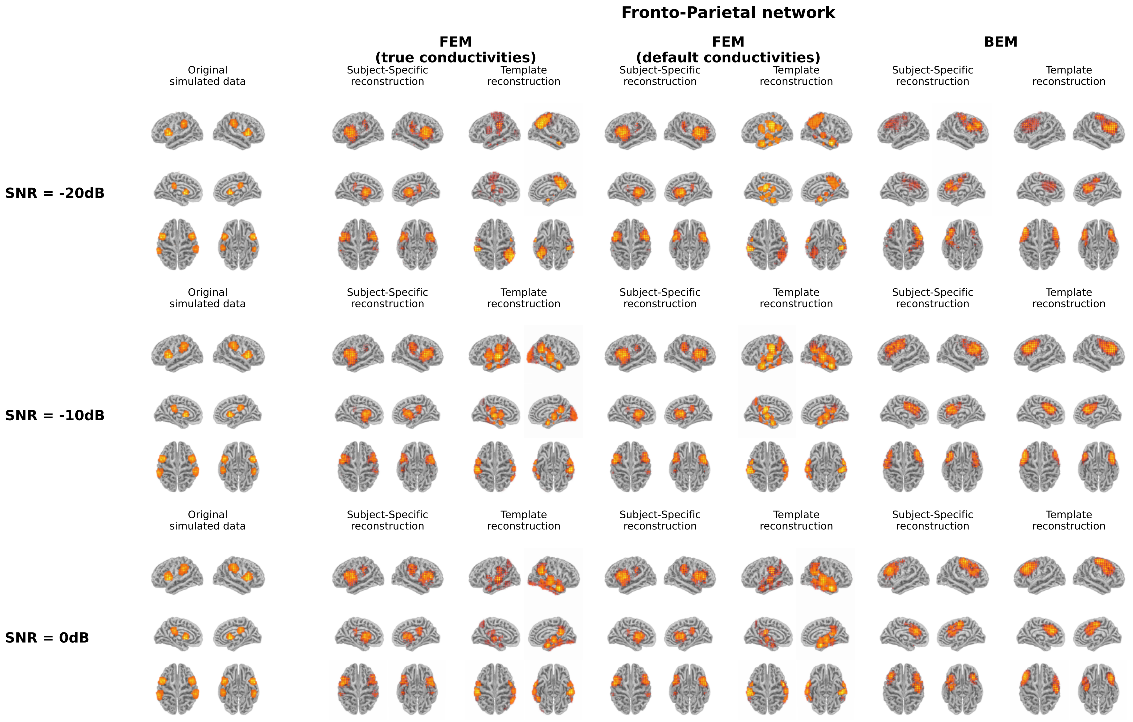

Figure S4. Overview of the original simulated data and the reconstructed activity averaged over all subjects for the fronto-parietal network at the different SNRs. In the case of the simulations and the subject-specific reconstructions, the source activity was morphed to the average head model before averaging.

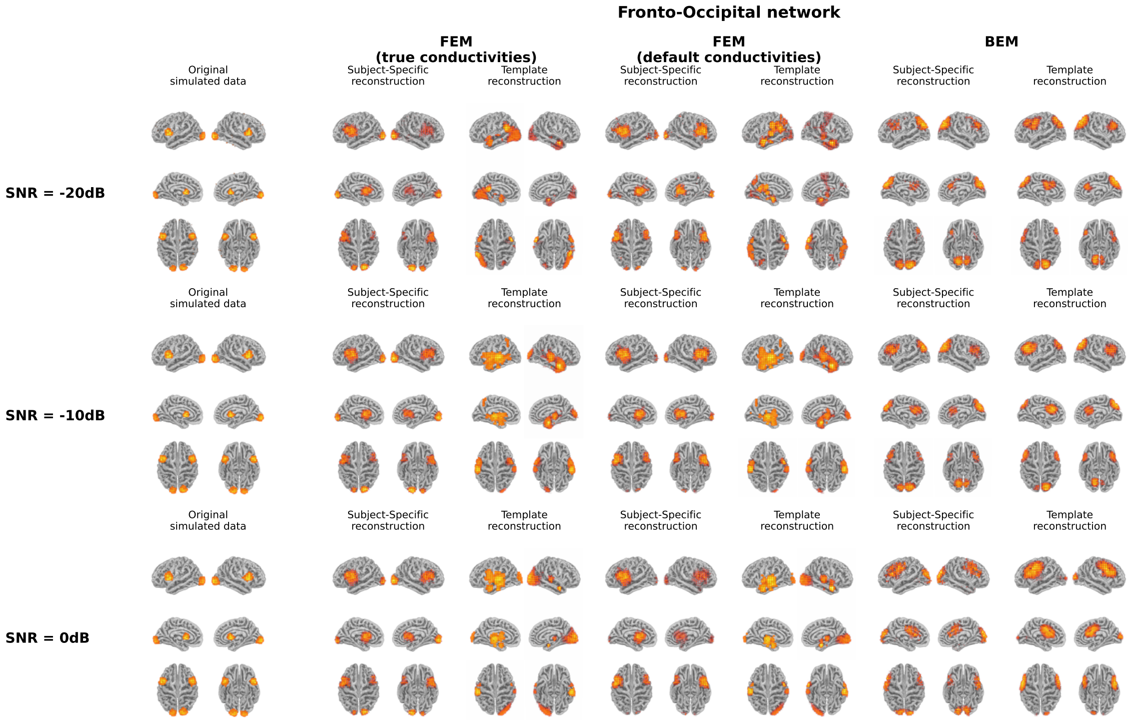

Figure S5. Overview of the original simulated data and the reconstructed activity averaged over all subjects for the fronto-occipital network at the different SNRs. In the case of the simulations and the subject-specific reconstructions, the source activity was morphed to the average head model before averaging.

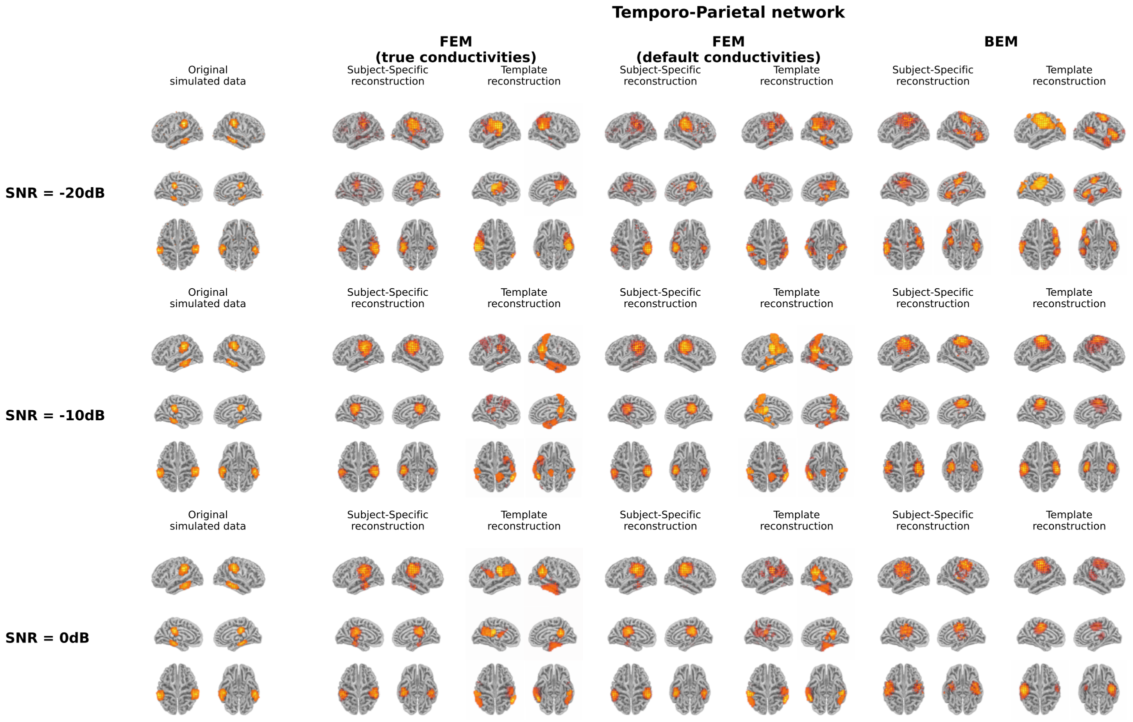

Figure S6. Overview of the original simulated data and the reconstructed activity averaged over all subjects for the temporo-parietal network at the different SNRs. In the case of the simulations and the subject-specific reconstructions, the source activity was morphed to the average head model before averaging.

APPENDIX C: QUANTIFICATION OF THE LOCALIZATION ERRORS WHEN USING DIFFERENT MAXIMAL DISTANCES OF 1 CM AND 5 CM AS THRESHOLD

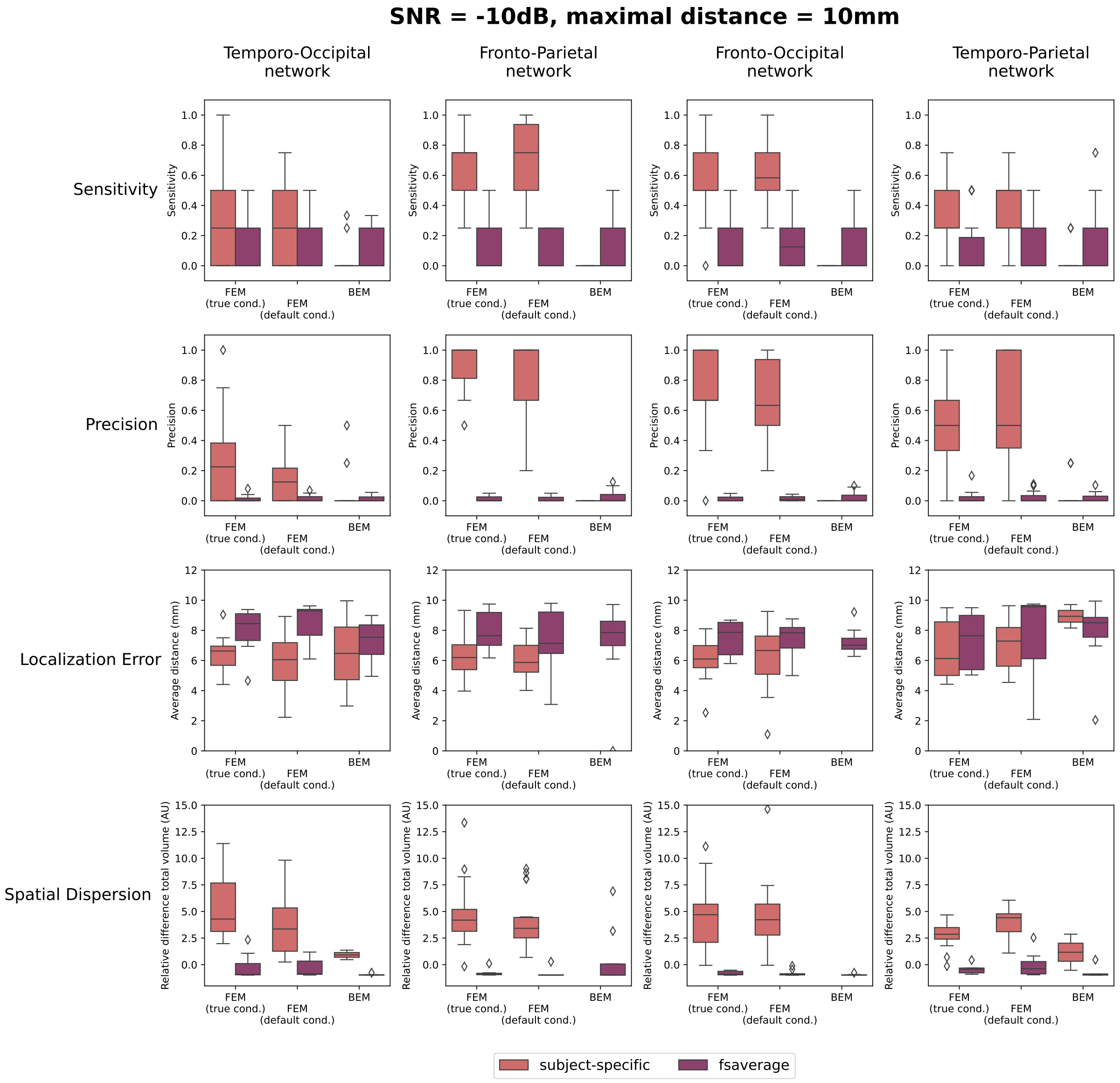

Figure S7. Results of the quantification of the localization errors. In this evaluation sensitivity and the precision of the obtained sources, the localization error and the spatial dispersion of these reconstructed sources were taken into account. For each of these measures, the difference between using the subject-specific and average head models is shown for each of the simulated networks. Clusters of activity were considered to be correctly localized when the difference between the center of the reconstructed cluster was within 1 cm of the center of the simulated ROIs.

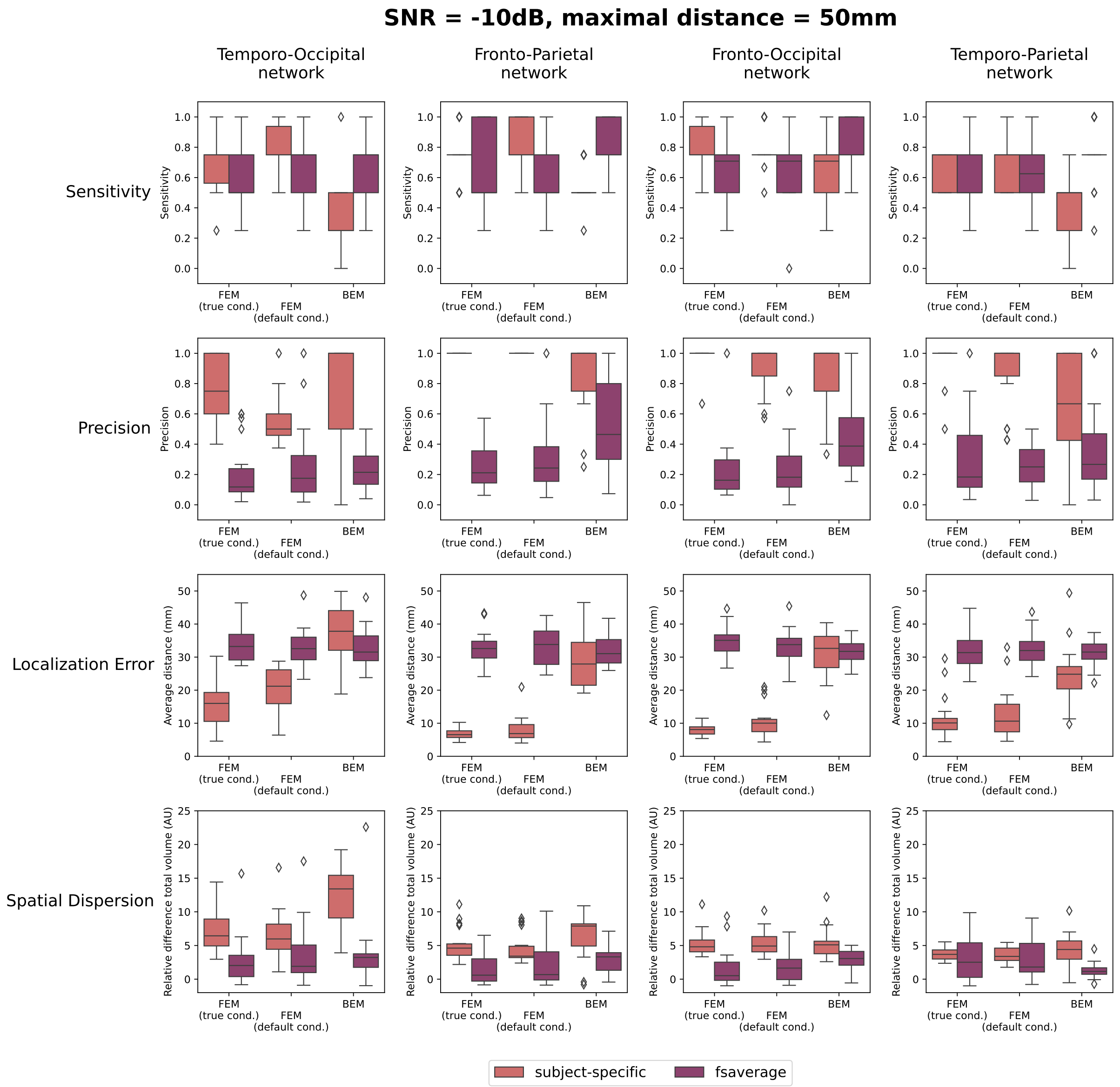

Figure S8. Results of the quantification of the localization errors. In this evaluation sensitivity and the precision of the obtained sources, the localization error and the spatial dispersion of these reconstructed sources were taken into account. For each of these measures, the difference between using the subject-specific and average head models is shown for each of the simulated networks. Clusters of activity were considered to be correctly localized when the difference between the center of the reconstructed cluster was within 5 cm of the center of the simulated ROIs.

APPENDIX D: QUANTIFICATION OF THE LOCALIZATION ERRORS WHEN USING DIFFERENT MAXIMAL DISTANCES OF 1 CM AND 5 CM AS THRESHOLD

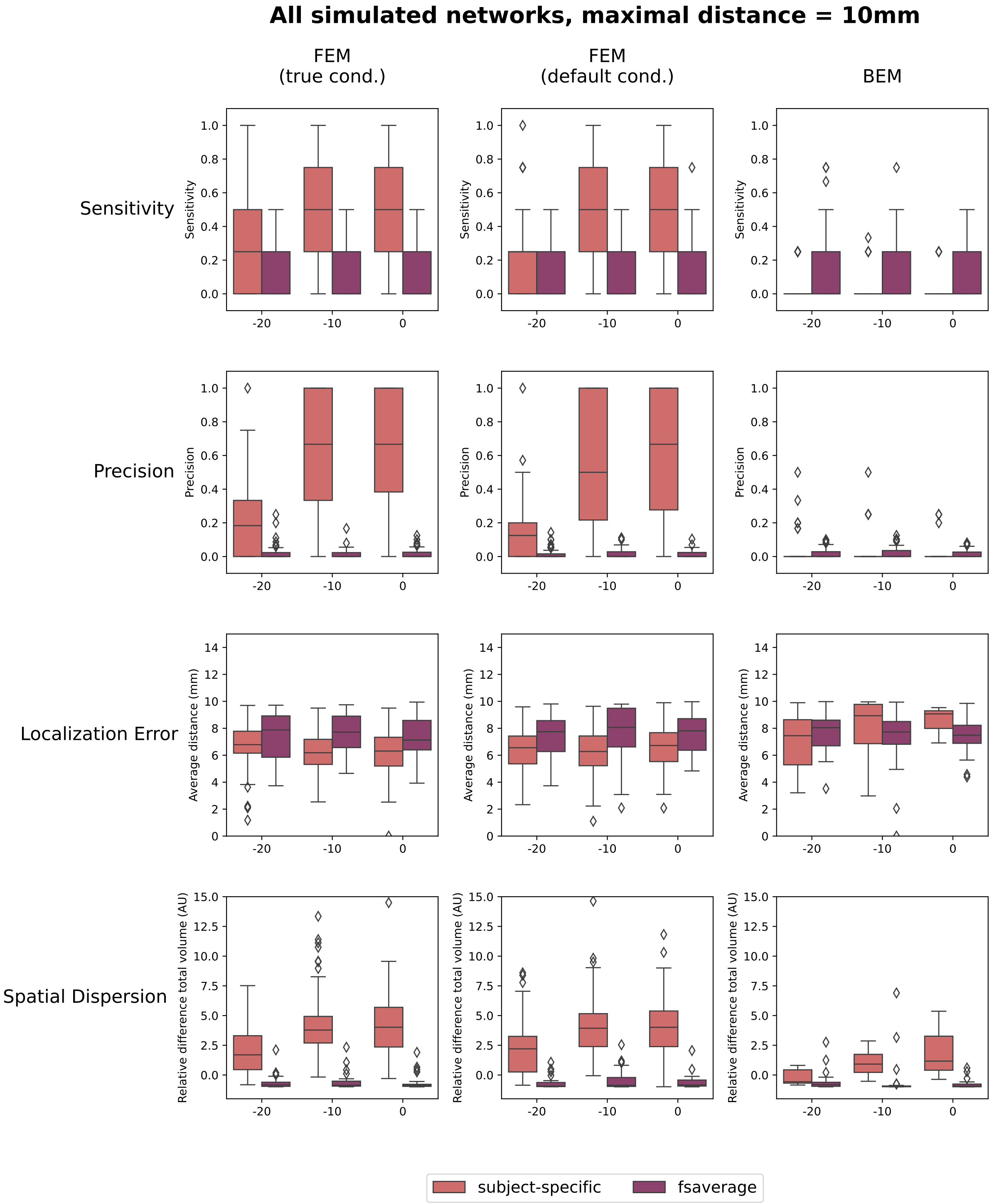

Figure S9. Results of the quantification of the localization errors. In this evaluation sensitivity and the precision of the obtained sources, the localization error and the spatial dispersion of these reconstructed sources were taken into account. For each of these measures, the effect of both the SNR of the simulated data and the difference between using the subject-specific and average head models is shown. Clusters of activity were considered to be correctly localized when the difference between the center of the reconstructed cluster was within 1 cm of the center of the simulated ROIs.

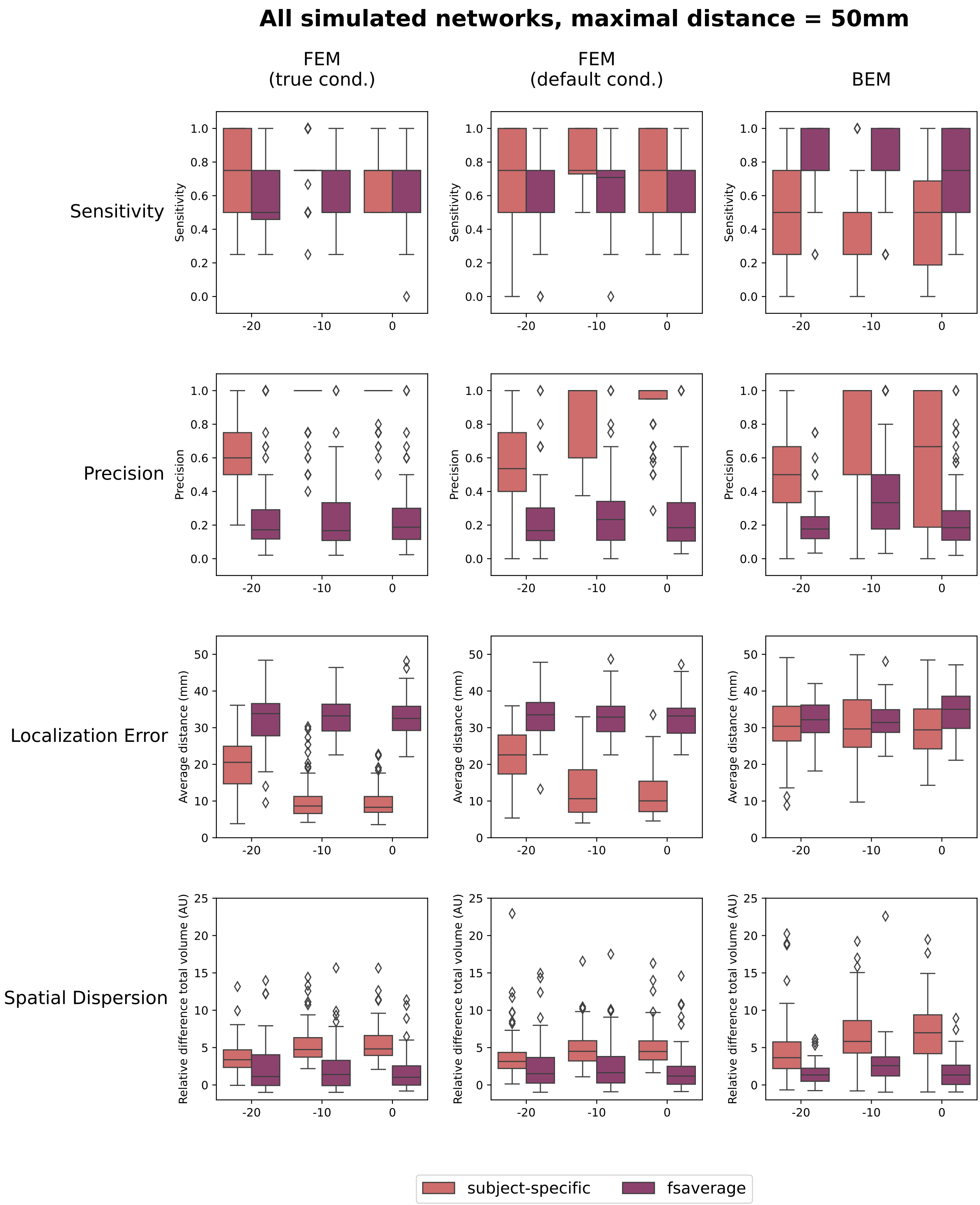

Figure S10. Results of the quantification of the localization errors. In this evaluation sensitivity and the precision of the obtained sources, the localization error and the spatial dispersion of these reconstructed sources were taken into account. For each of these measures, the effect of both the SNR of the simulated data and the difference between using the subject-specific and average head models is shown. Clusters of activity were considered to be correctly localized when the difference between the center of the reconstructed cluster was within 5 cm of the center of the simulated ROIs.
